# Effects of edible bird’s nest and EDTA on cadmium toxicity exposed rats’ embryo production, quality, and pre- and post-embryo transfer pregnancy rates

**DOI:** 10.5455/javar.2024.k844

**Published:** 2024-12-29

**Authors:** Anmar Jasim Mohammed, Nurhusien Yimer, Faez Firdaus Abdullah Jesse, Wan Nor Fitri Wan Jaafar, Ainu Husna

**Affiliations:** 1 Department of Veterinary Clinical Studies, Faculty of Veterinary Medicine, University Putra Malaysia, Seri Kembangan, Malaysia; 2 College of Veterinary Medicine, University of Fallujah, Fallujah, 31002, Iraq; 3 Department of Veterinary Sciences, School of Medicine, International Medical University, Kuala Lumpur, Malaysia; 4 Division of Veterinary Reproduction, Faculty of Veterinary Medicine, Universitas Airlangga, Surabaya, Indonesia; 5 Farm and Exotic Animal Medicine and Surgery, Faculty of Veterinary Medicine, University Putra Serdang, Seri Kembangan, Malaysia; 6 Livestock Science Division, Malaysian Agricultural Research Institute, Serdang, Selangor 43400, Malaysia

**Keywords:** Embryo transfer, Edible bird’s nest, Cadmium toxicity, pregnancy, blastocyst

## Abstract

**Objectives:**

The current study aimed to investigate the prophylactic potential of EBN compared to EDTA in mitigating Cd's toxic effects on pregnancy rates and embryonic development in rats.

**Materials and Methods:**

Ninety-eight female rats (Sprague Dawley) were divided into donor and recipient groups, with donors further divided into seven subgroups, including negative control, Cd-exposed, EBN-treated, and EDTA-treated groups. Embryos from donors were transferred to recipient rats, with EBN and Cd administered for 4 weeks and EDTA given only in the last 5 days for the donor group.

**Results:**

Results showed significant differences in pregnancy rates and blastocyst quality. EBN at 120 mg/kg BW led to higher blastocyst production and better quality compared to Cd-exposed groups. The highest pregnancy rates in recipient groups correlated with the highest blastocyst scores from donors.

**Conclusion:**

EBN at 120 mg/kg demonstrated significant protection against Cd toxicity and its effect on pregnancy rates, embryo production, quality, and pre- and post-embryo transfer, surpassing the effects of both 90 mg/kg EBN and EDTA. This study provides empirical evidence in support of the conventional belief in the positive impact of EBN on female reproduction.

## Introduction

Edible Bird's Nest (EBN) is cup-shaped, usually white, yellow, or red, and is mainly made from the salivary secretion of male swiftlets [1]. It is a popular ingredient in traditional Chinese medicine and cuisine, mainly comprising glycoproteins; its nutritional composition includes protein, carbohydrate, ash, and lipid in descending order of concentration [2]. EBN analysis has also revealed the presence of various substances, such as vitamins and mineral elements [3], along with reproductive hormones such as testosterone, estradiol, progesterone, LH, FSH, and prolactin [1]. Evidence from research studies showed that EBN exerts immunostimulatory activity, induces cell division and proliferation, inactivates the influenza virus, and is implicated in the amelioration of osteoporosis conditions [4,5]. Although several previous studies have investigated the different non-reproductive biological activities of EBN, such as its potent antioxidant activity [6]. Recent interest has increasingly been focused on its potential roles in reproductive functions. The presence of reproductive hormones, in addition to their antioxidant effects, gives promising avenues for *in vivo* and *in vitro* applications with respect to reproductive health by reducing oxidative stress.

Cadmium (Cd) is a widely distributed and highly toxic transition metal in the environment, potentially exposing the general population through various pathways, including food, water, inhalation, and tobacco smoke [7]. Cd is a nonessential element that is toxic at low doses and is resistant to biodegradation with a long biological half-life. Cd accumulates in organs like the lung, gut, liver, kidneys, placenta, mammary glands, uterus, and fetus upon exposure [8]. The detrimental effects of heavy metals include osteomalacia, hepatotoxicity, renal toxicity, neurotoxicity, infertility, and cancer [9]. Cd induces oxidative stress by depleting glutathione and sulfhydryl groups, leading to increased reactive oxygen species production. In reproductive contexts, Cd is associated with reduced embryo quality, early developmental block, fragmentation, lower blastulation rate, and decreased pregnancy rates [10]. Cd is known to be an endocrine disruptor. Cd exposure suppresses pituitary hormones in rodents; gonadotropins, prolactin, ACTH, growth hormone, and thyroid-stimulating hormone [11]. Oocyte maturation in the ovary has been disturbed upon Cd treatment in different species; among them are rats and mice [12].

Embryo transfer (ET), a crucial step in assisted reproduction, involves placing embryos into a female's uterus to establish pregnancy. Walter Heape achieved the first successful mammalian ET in 1890, transferring Angora rabbit embryos into a Belgian doe [13]. In laboratories, ET is employed in genetically modified mice and rats. Genetically modified mouse embryos can be frozen and implanted into a surrogate dam when needed. Rats, widely used in biomedical research, offer simplicity and size advantages. Comprehensive databases like the National BioResource Project-Rat in Japan and the Rat Genome Database in the USA support rat-related research [14,15]. Modern practices involve using supplements, including proteins and amino acids, to enhance embryo quality [16]. However, only a few studies have investigated the role of EBN in improving fertility, uterine function, and embryo implantation rates [17].

This study evaluates the role of *in vivo* EBN supplementation versus EDTA in providing prophylaxis against Cd toxicity on embryo quality, embryo production, and levels of the pregnancy hormone progesterone in the 1st and 2nd trimesters of pregnancy, as well as pre- and post-ET pregnancy rates.

## Material and Methods

### Ethical approval

The animals were treated humanely, and their care was in accordance with the guidelines of the Committee for Institutional Animal Care and Use Committee UPM/IACUC/AUP-R037/2022, Universiti Putra Malaysia (UPM).

### Animals and experimental designs

Twelve-week-old female rats (Sprague Dawley) were purchased from the Forensic Alchemist Resources and Trading Centre, Selangor, and adapted for 7 days. The rats were kept in cages with unrestricted access to food (Gold Coin Feed Mills, Malaysia) and water. They were provided humane care and housed following the guidelines set by the Committee for Institutional Animal Care and Use (UPM/IACUC/AUP-R037/2022) at UPM. Throughout the adaptation period, vaginal cytology was used to confirm the regularity of the estrous cycles. Following this period, the rats were randomly assigned to two groups: donors (7 groups, six animals per group, and treated as the following: T0 = Negative control, T1 = Cd 5 mg/kg BW, T2 = EBN 120 mg/kg BW, T3 = EDTA 0.5 mmol/kg BW, T4 = Cd 5 mg/kg BW + EBN 120 mg/kg BW, T5 = Cd 5 mg/kg BW + EBN 90 mg/kg BW, T6 = Cd 5 mg/kg BW + EDTA 0.5 mmol/kg BW (Table 1). Recipient groups are also subdivided into seven groups (eight animals per group). The recipient group did not receive any treatment, which is only used in ETs. Donor groups were exposed to CdCl₂ (5 mg/kg BW) [18], EBN supplements for 4 weeks, and EDTA 0.5 mmol/kg [19].

### EBN preparation, composition, and usage

A hydrolyzed form of EBN obtained from Agridon Technologies, Malaysia (House Bird's Nest) through the National University of Malaysia was used in the present study. Production of EBN started with cleaning the raw EBN by soaking it in water for 4–8 h to rehydrate. Upon rehydration, feathers and other debris like dirt were removed using tweezers, and EBN was washed profusely with clean water [20]. Next, the EBN was prepared for hydrolysis by cutting or tearing it into smaller pieces to increase the surface area for enzyme action. An enzyme solution was prepared by making a solution of the chosen enzyme in water, such as pepsin, trypsin, or papain, according to the instructions of the manufacturer. The solution was adjusted to the optimum pH range of the enzyme used: pepsin in the pH range 2–3, trypsin in the pH 7–8 range, and papain in the pH 6–7 range—using hydrochloric acid or sodium hydroxide, respectively. Subsequently, EBN pieces were added to the enzyme solution and incubated for 4–6 h at the optimum temperature of the enzyme (37°C for pepsin, 50°C for trypsin, and 40°C–60°C for papain). This hydrolysis was then stopped by heating the mixture to approximately 90°C for 10–15 min to inactivate the enzyme [21]. Accompanied by the obtained hydrolyzed EBN was a certificate of its composition analysis with 9.7% sialic acid, 58.9 gm/100 gm protein, 0.1 gm/100 gm fat, 26.2 gm/100 gm carbohydrate, and 2.2 gm/100 gm ash.

**Table 1.** Animal grouping and feeding regime.

Group	Grouping	Type of feed (dose) and Cd dose
Negative Control	T0	Normal diet (ND) + Distilled water given Orally - Distilled water given IP
Positive Control	T1	Cd only (5 mg/kg body wt., Orally) + Distilled water IP
	T2	EBN only (120 mg/kg BW. Orally)
	T3	EDTA (0.5 mmol/kg BW. IP)
Experimental group	T4	Cd + EBN (5 mg/kg body wt., Orally + 120 mg/kg BW IP)
	T5	Cd + EBN (5 mg/kg body wt., Orally + 90 mg/kg BW. orally)
	T6	Cd + EDTA (5 mg/kg body wt., Orally + 0.5 mmol/kg BW. IP)

ND = Normal diet, Cd = Cadmium Chloride, EBN = Edible bird’s nest, BW = Body weight, IP = Intraperitoneal.

Hydrolyzed EBN in its powder form (powdered using a BUCHI-400 mixer from Switzerland) was stored at a temperature of 4°C. To prepare the EBN solution, a modified version of the Chinese tradition of making bird's nest soup was employed. The EBN solution was prepared by dissolving 1 gm of hydrolyzed EBN powder in 100 ml of distilled water. This solution was then heated in a water bath at 60°C for 45 min. After that, the solution was cooled to room temperature and given to the rats orally using a gavage needle, according to the animal grouping [22].

### Preparation of cadmium chloride and EDTA solution

Cadmium chloride (CdCl₂), with a molecular weight of 183.32 g/mol, was purchased from R&K Chemicals, UK (CAS NO: 35658-65-2). CdCl₂ was dissolved in distilled water and delivered via oral gavage using a curved needle (18 G, 4.5 cm). The rats were calmly restrained to facilitate the insertion of the gavage needle. The needle was carefully placed in the left side of the mouth and gently slid down to the esophagus at the appropriate distance. CdCl₂ was administered to the rats for 4 weeks at a dose of 5 mg/kg body weight [22]. CaNa_2_EDTA solution was purchased from BRNSON Chemicals (USP) UK in a vial and injected directly by the intraperitoneal route.

### Preparation of vasectomized rats

**Figure 1.**
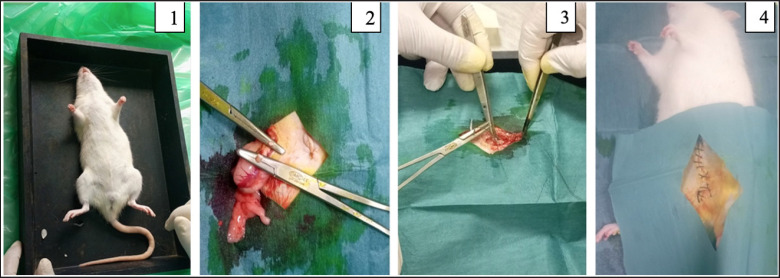
Rat vasectomy operation. (1) Male rat under general anesthesia (2) Cauterization and cutting of the vas deferens (3) Muscle suturing (4) Skin suturing.

The male rats (9 healthy males) were restrained under general anesthesia, achieved through the injection of combinations of 30 mg ketamine/kg body weight and 10 mg xylazine/kg body weight. Subsequently, the lower abdomen was wiped with chlorhexidine–alcohol 70%. Vasectomy was then carried out according to Timurkan [23]. Abdominal surgery involved making two 1-cm longitudinal incisions through the skin and abdominal wall on each side. The testes were exteriorized, the vasa deferentia located, and a small section was ablated using hot thumb forceps. The testes were then returned to the abdomen. The incisions in the abdominal wall were closed using Vicryl 5.0 sutures (Johnson & Johnson, New Brunswick, NJ, USA) (Fig. 1). Finally, the vasectomized rats were housed individually in cages. After 3–4 weeks, they were used to produce pseudo-pregnant rats.

### Preparation of pseudo-pregnant (recipient) female rats

Fifty-eight female rats were used to prepare pseudopregnant rats as recipients. To synchronize the rats, two intraperitoneal injections (0.2 ml) of PGF2α (Estrumate, Merck, GmbH) were administered per rat, each containing 0.5 mg of cloprostenol, with a 3-day interval between doses [24]. Following synchronization, the rats were paired with vasectomized male rats in a one-to-one ratio, and mating was confirmed by the presence of a copulatory plug in the vagina.

### Donor preparation and embryo collection

In the final 5 days of the treatment, healthy and fertile male rats were introduced into the donor group at a one-to-one ratio. The mating status of the females was confirmed by the presence of a copulation plug. On the last day of the experiment (4 days after mating), embryo harvesting was performed. The female donor rat was euthanized following the approved ethics committee protocol, and each uterine horn with the attached oviduct was carefully removed, minimizing the presence of fat and blood vessels. The uterine horns were collected in an M2-filled tissue culture dish, rinsed, and additional debris was delicately removed using a tissue. The uterine horn was rolled on the tissue to further eliminate blood, placed in a 60 mm tissue culture dish, and observed under a dissecting scope while being flushed with media using a 26-gauge needle. The goal was to keep flushes in discrete drops for simple identification of embryos. This process was repeated for the second uterine horn, and embryos were collected in M2 using an ET pipette as outlined by Rozkova [25] with some modifications.

### Embryo transfers with the rNSET device

Female pseudopregnant recipients were given an intraperitoneal injection of 2 IU of oxytocin (Oxoject, Covetrus, North America) 2 h before ET. The embryos were prepared for transfer in a 70 μl drop of M2 medium (EmbryoMax, Merck Chemicals GmbH). The rNSET device (ParaTechs, Lexington, KY) was attached to a Rainin PR-2 Pipette (Mettler-Toledo Rainin, Oakland, CA) set to 1.8 µl, and the embryos were loaded into the rNSET device. The catheter of the rNSET device was then inserted through the speculum, past the cervix, and into the uterine horn following the administration of Acepromazine (1.0–2.5 mg/kg, IM) to mildly sedate the rat [26,27]. At the end of the experiment, the pregnancy rate in recipient rats was confirmed by observing the successful delivery of pups.

### Assessment of rats' embryo quality

The blastocyst scoring system included an assessment of the development stage, along with the grading of the inner cell mass (ICM) and trophectoderm (TE) [28]. For example, a blastocyst in the expanded stage with a good ICM and a fair TE is scored as 3AB. This numerical interpretation aligned with Gardner [29]. It was observed that if a blastocyst had collapsed during the assessment, reliable grading was not possible. Such blastocysts required re-evaluation 1–2 h later, as normal cycles of collapse and re-expansion were part of the blastocyst's natural progression. To enable comparisons with the current study’s results, the blastocysts were given a four-character score based on their degree of expansion and the grades of their ICM and TE, just before transfer. The scores categorized the blastocysts as “excellent” (≥3AA), “good” (3–6AB, 3–6BA, 1–2AA), “average” (3–6BB, 3–6AC, 3–6CA, 1–2AB, 1–2BA), or “poor” (1–6BC, 1–6CB, 1–6CC, 1–2BB) [30].

### Plasma progesterone assay

The progesterone ELISA kit was used according to the procedure provided by the manufacturer (Qayee Bio-Technology Co., China) and published by Quddus [22].

### Statistical analysis

The Statistical Analysis System (SAS, 2022) software was employed to assess the impact of various factors on the study parameters. The Least Significant Difference test, following Analysis of Variance, was used to compare mean differences for statistical significance. The chi-square test was applied to compare percentages. Differences were considered significant if the probability (*p*) was less than 0.05 or 0.01 [31].


$$\chi^{2}\;=\;\sum \;\frac{(O\;–\;E)2}{E}$$

χ^2^: *Chi*-square, Σ: Summation, O: Observed No., E: Expected No.

## Results

### Pregnancy rate in donor rats

**Figure 2.**
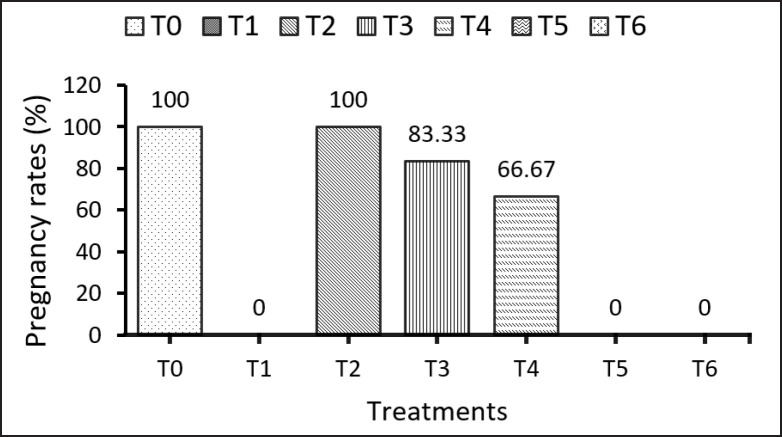
Effects of EBN and EDTA on pregnancy rates of donor rats subjected to Cd toxicity. Note: T0 = Negative control, T1 = Cd 5 mg/kg BW, T2 = EBN 120 mg/kg BW, T3 = EDTA 0.5mmol/kg BW, T4 = Cd 5 mg/kg BW + EBN120 mg/kg BW, T5 = Cd 5 mg/kg BW + EBN90 mg/kg BW, T6 = Cd 5 mg/kg BW + EDTA 0.5 mmol/kg BW.

The administration of (Cd) for 4 weeks notably impacted the pregnancy rate. Figure 2 illustrates that Cd toxicity significantly reduced the likelihood of pregnancy in groups T1, T5, and T6. However, the administration of EBN at a dosage of 120 mg/kg BW. mitigated the toxic effects of Cd, as evidenced by the improved pregnancy rate observed in group T4 (Fig. 2). It was observed that the administration of EBN or EDTA alone did not significantly affect the pregnancy rate compared to the control group.

### Harvested blastocysts from donor groups

As shown in Table 2, the administration of Cd for 4 weeks significantly affected the number of blastocysts. In T1, which was treated with Cd alone, the production of blastocysts was prevented. Additionally, groups T5 and T6 also exhibited zero blastocyst production. Conversely, the T2 group, treated with a combination of Cd and 120 mg/kg BW of EBN, demonstrated that EBN at a dose of 120 mg/kg BW can reduce the toxic effects of Cd as shown in Table 2.

The percentage of blastocysts produced by the donor group is calculated using the following formula:

The mean of produced blastocysts/No. of pregnant animals * 100%

It was observed that the administration of EBN or EDTA alone did not significantly affect the percentage of blastocyst production compared to the control group.

### Assessment of rats' embryo quality

**Figure 3.**
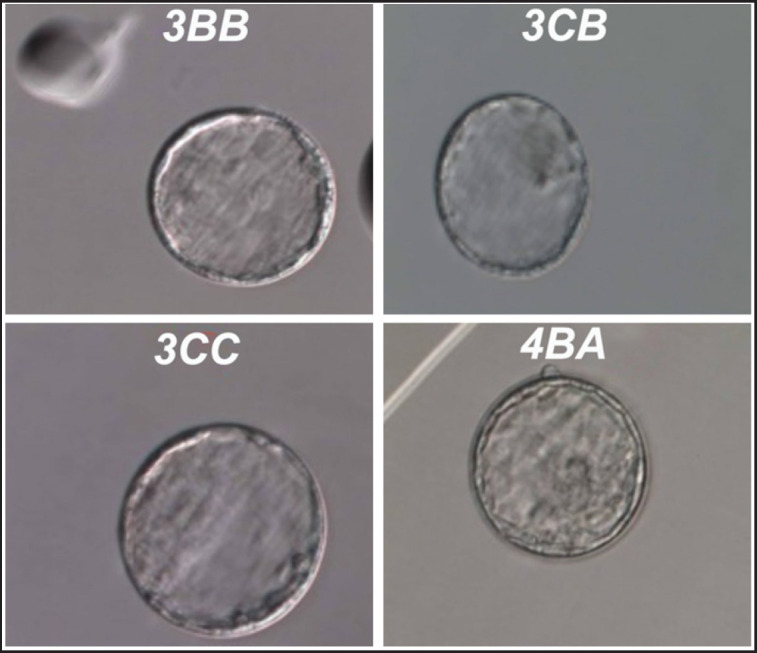
Blastocyst in different scores. 3CC Full blastocyst, the blastocyst cavity fills the embryo, very few inner cells mass, and the Trophectoderm contains very few cells forming a loose epithelium. 4BA Expanded blastocyst, the blastocyst cavity volume is larger than that of the early embryo and the surrounding membrane is thinning with loosely grouped, several inner cell masses and many cells forming a tightly knit epithelium of Trophectoderm. 3BB Full blastocyst, the blastocyst cavity fills the embryo loosely grouped, several inner cell mass with few cells of Trophectoderm. 3CB Full blastocyst with Very few inner cells mass and few cells of Trophectoderm.

Representative blastocyst-quality pictures are shown in Figure 3. The quality of the blastocyst of all groups (T0, T2, T3, and T4) is depicted in Table 3. The result showed that there is a significant difference in excellent scores between all groups at *p* < 0.05, but not in the other scores. T2 (EBN 120 mg/kg bw) resulted in the highest percentage of excellent blastocyst scores compared to the other groups. No significant differences were observed among other groups in the grading of the blastocyst (pregnant groups).

**Figure 4.**
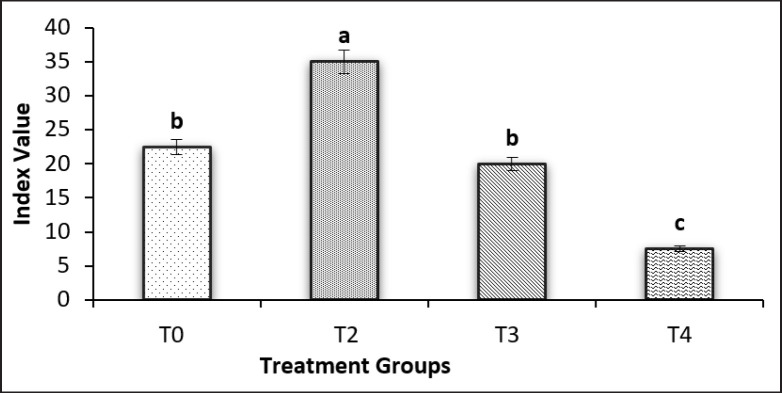
Index value for blastocyst production in different groups. Note: T0 = Negative control, T2 = EBN 120 mg/kg BW, T3 = EDTA 0.5 mmol/kg BW, T4 = Cd 5 mg/kg BW + EBN120 mg/kg BW.

The index value for blastocyst production in different groups showed significant differences in all groups, represented by the highest production in group T2, which was treated with EBN 120 mg/kg BW, while the lowest production was observed in the T4 group, which was treated with EBN 120 mg/kg BW and Cd 5 mg/kg BW (Fig. 4).

### Pregnancy rates among recipient groups

The percentages of pregnant rats within the recipient groups were influenced by the quality of the blastocyst. All groups exhibited highly significant differences (*p* < 0.01). Specifically, the T2 group, which received three blastocysts per animal with higher scores (excellent), demonstrated the highest pregnancy percentage compared to the other groups, as illustrated in Table 2.

### Plasma concentration of progesterone in the 1st and 2nd trimesters of pregnancy

In the 1st trimester, on the fourth day of pregnancy, plasma progesterone level was reduced by Cd 5 mg/kg BW, Cd 5 mg/kg BW + EBN 90 mg/kg BW, and Cd 5 mg/kg BW + EDTA 0.5 mmol/kg BW. Progesterone concentration in T1, which underwent treatment with only Cd, showed a dramatic decrease compared to that of the control group. In contrast, progesterone concentrations in the T4 group (EBN 120 mg/kg BW) increased significantly compared to the control group. During the 2nd trimester of pregnancy, the progesterone level in recipients without treatment was high only in the T2 group, which received embryos from the group treated with EBN 120 mg/kg BW.

**Table 2.** Percentage of blastocyst production by donor groups and pregnant animals in recipient group.

Doner Group				Recipient group	
Group	No. of pregnant animals	Mean ± SE	Percentage of blastocyst % Doner Group	No. of recipient Animals	Percentage of pregnant animals
T0	5	3.6 ± 0.81	72%	8	62.5%
T1	0	0	0	0	0
T2	6	4.2 ± 0.74	70%	8	87.5%
T3	5	3.4 ± 0.6	68%	8	50%
T4	4	2 ± 0.4	50%	8	37%
T5	0	0	0	0	0
T6	0	0	0	0	0
Chi-square: χ^2^ (*p*-value)	---	---	8.483 ** (0.0071)	---	8.921 ** (00068)

** (*p *≤ 0.01). Note: T0 = Negative control, T1 = Cd 5 mg/kg BW, T2 = EBN 120 mg/kg BW, T3 = EDTA 0.5 mmol/kg BW, T4 = Cd 5 mg/kg BW + EBN120 mg/kg BW, T5 = Cd 5 mg/kg BW + EBN90 mg/kg BW, T6 = Cd 5 mg/kg BW + EDTA 0.5 mmol/kg BW. (T represents the groups which blastocysts came from. All Recipient animals did not receive any treatment).

**Figure 5.**
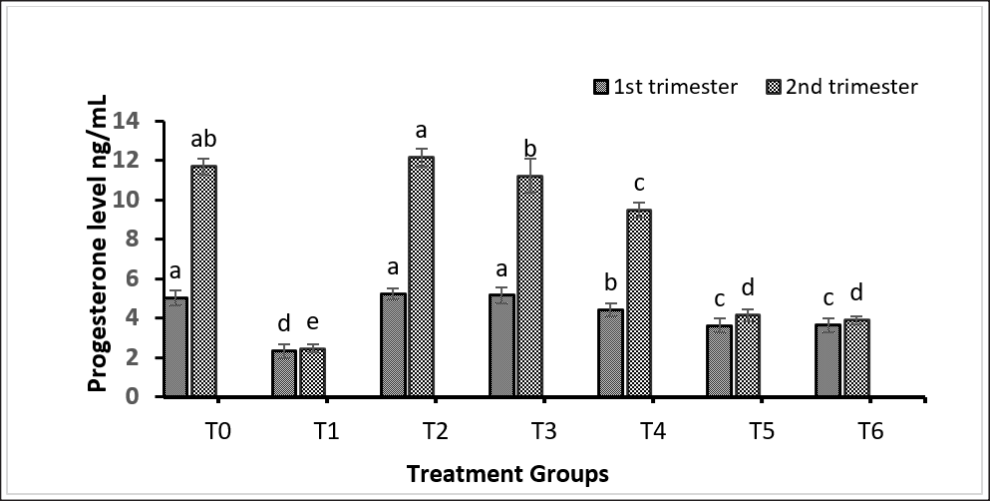
Progesterone levels during 1^st^ trimester of pregnancy in donor groups and 2^nd^ trimester of recipient groups. Note: T0 = Negative control, T1 = Cd 5 mg/kg BW, T2 = EBN 120 mg/kg BW, T3 = EDTA 0.5 mmol/kg BW, T4 = Cd 5 mg/kg BW + EBN120 mg/kg BW, T5 = Cd 5 mg/kg BW + EBN90 mg/kg BW, T6 = Cd 5 mg/kg BW + EDTA 0.5 mmol/kg BW.

In the T4 group, levels were lower in the treatment of EBN 120 mg/kg BW + Cd 5 mg/kg BW in comparison with both the control group and the T3 group treated with 0.5 mmol/kg BW of EDTA. Other groups showed low progesterone levels similar to those of the non-pregnant rats (Fig. 5).

## Discussion

The findings of the current study elucidate a pronounced negative impact of Cd on the pregnancy rate following a 4-week administration. These observations corroborate with the research conducted by Zhu [32]*, *wherein it was noted that Cd exposure in female mice induces deleterious effects on the meiotic maturation of oocytes and subsequent embryonic development. The underlying mechanisms for this impairment were attributed to the detrimental influences of Cd on cytoskeletal organization, mitochondrial function, and histone modifications. In essence, the deleterious influence of Cd on these pivotal cellular processes collectively contributes to a discernible reduction in the pregnancy rate.

Cd toxicity adversely affects reproduction, classified as an endocrine disrupter [33,34,12]. Female reproductive system studies confirm harmful effects in both short- and long-term exposures [35–37]. Recent research highlights Cd accumulation in females, crossing the placental barrier, and impacting fetal development [38]. Cd nanoparticles, crossing the placenta, detrimentally affect reproductive success and perinatal growth [8,39]. Cd's impact on mammalian health is intricate due to its mimicry of essential metals [40].

Various compounds, including those from chemicals, plants, and animals, protect against heavy metal toxicity [41]. EBN, made from swiftlet birds' saliva, is recognized for its potential as a hormone replacement prophylaxis without reported side effects [17,42]. Studies suggest EDTA's superiority over DMSA in mobilizing intracellular Cd, with FDA approval for treating heavy metal toxicity [43,44].

**Table 3.** Grading of blastocyst for different groups.

Group	Excellent %	Good %	Average %	Poor %	Chi-square: χ2 (*p-value)*
T0	22.22	33.34	16.6	27.9	1.11 NS (0.774)
T2	36	28	16	20	2.360 NS (0.501)
T3	17.6	23.51	29.41	35.29	1.11 NS (0.774)
T4	16.6	33.3	33.3	16.4	0.667 NS (0.881)
Chi-Square: χ^2^ (*p*-value)	8.176 * (0.042)	3.105 NS (0.375)	1.428 NS (0.698)	3.470 NS (0.324)	

* (p ≤ 0.05), NS: Non-Significant. Note: T0 = Negative control, T1 = Cd 5 mg/kg BW, T2 = EBN 120 mg/kg BW, T3 = EDTA 0.5 mmol/kg BW, T4 = Cd 5 mg/kg BW + EBN120 mg/kg BW, T5 = Cd 5 mg/kg BW + EBN90 mg/kg BW, T6 = Cd 5 mg/kg BW + EDTA 0.5 mmol/kg BW.

In the present study, EBN administration markedly enhanced the percentage of pregnant female donors. Furthermore, EBN (120 mg/kg BW) exhibited a pronounced capacity to mitigate the adverse impact of Cd on the pregnancy rate. This finding aligns with the results of a study conducted by Albishtue [17], wherein EBN was observed to exert a promotive effect on fertility indexes, specifically by augmenting pregnancy rates.

It is well-established that cadmium (Cd) exhibits high toxicity towards the developing embryo. However, emerging evidence suggests that Cd, along with nickel and other heavy metals, also acts as a developmental toxicant. Specifically, exposure to Cd during early embryonic stages has been associated with a reduction in blastocyst formation.

Our investigation revealed that a four-week exposure to Cd significantly impacts the number of blastocysts (T1). These results agree with those of a study by Zhao [30], whereby they investigated the effects of Cd on early embryonic development *in vitro*. In that previous study, with an increased concentration of Cd, there was a significant reduction in the production and formation rate of the blastocyst in mice.

In the present study, EBN was shown to enhance blastocyst production; this is consistent with Albishtue [17], who noticed improved P4 levels and growth of ultra-structural pinopods involved in embryo attachment to the uterine epithelium. Quddus [22] found EBN to play a role in maintaining the level of progesterone exposed to cadmium in rats. Progesterone participates in both embryo development and implantation via endometrial receptivity, embryonic survival through gestation, and change of endometrial stromal cells to decidual cells. In their study, Lonergan and Sánchez [45] went further to add that progesterone affects the process of oocyte maturation and embryo development.

It is also important to maintain an optimal balance of progesterone for the successful formation of a blastocyst. Too low a level of progesterone may affect the endometrial environment and thereby limit the implantation and development process of the blastocyst, and too high a level of progesterone could also disturbingly affect the production of the blastocyst [46]. Especially about this view, our research also showed that the number of blastocysts in the group treated by Cd 5 mg/kg BW + EBN 120 mg/kg BW was higher than in the group treated by Cd 5 mg/kg BW alone. On the contrary, the production of blastocysts was completely absent in groups treated with Cd 5 mg/kg BW + EBN 90 mg/kg BW and Cd 5 mg/kg BW + EDTA 0.5 mmol/kg BW, respectively, and is correlated to low levels of progesterone.

Progesterone is one of the major female reproductive hormones found to play an extremely essential role in fertility maintenance. As explained earlier, progesterone takes part in the development and differentiation of reproductive tissues; its reduced synthesis could be caused by this specific inhibition of steroidogenic genes, resulting from cadmium exposure [47]. Exposure of Cd in cultured ovarian granulosa cells has been linked to decreased progesterone synthesis, concurrent with morphological changes. Cadmium-induced damage in the ovarian tissues can cause hormonal imbalance, hence affecting ovarian function, a system in charge of producing hormones like progesterone [48].

Maintaining progesterone levels is critical for successful embryonic development and subsequent pregnancy [49]. Our findings align with these observations, indicating the lowest progesterone levels in groups treated with Cd 5 mg/kg BW, Cd 5 mg/kg BW + EBN 90 mg/kg BW, and Cd 5 mg/kg BW + EDTA 0.5 mmol/kg BW, while the highest levels were observed in the control group, EBN 120 mg/kg BW, and EDTA 0.5 mmol/kg BW-treated groups. This significant difference was noted in comparison to the treatment with T4, which was treated with Cd 5 mg/kg BW + EBN 120 mg/kg BW, observed during the 1st and 2nd trimesters of pregnancy. Similar results regarding progesterone levels during exposure to Cd and EBN were reported by Quddus [22].

The current study has revealed a noteworthy disparity in the type of blastocysts harvested with excellent score quality among all groups, while no such distinctions were observed in the other scores. The most favorable outcome was observed in the group treated solely with EBN at a dosage of 120 mg/kg BW. Except for groups that failed to produce blastocysts, such as groups treated with Cd 5 mg/kg bw, Cd 5 mg/kg BW + EBN 90 mg/kg BW, and Cd 5 mg/kg bw + EDTA 0.5 mmol/kg bw, the least favorable result was noted in the group treated with Cd 5 mg/kg BW + EBN 120 mg/kg BW. These notable differences in the effects are probably due to the accumulation of Cd in embryos from the four-cell stage onward. Higher exposure levels prevent development to the blastocyst stage and cause degeneration and decompaction of blastocysts after their formation. The quality of embryos may be impaired by apoptosis and disruption of cell adhesion that results from these detrimental effects. These findings align with the results reported by Thompson and Bannigan [34]. Cd is recognized for inducing oxidative stress through the production of reactive oxygen species (ROS). The action of increased levels of ROS causes irreversible cellular damage to DNA, proteins, and lipids in developing embryos, which can affect the quality of the blastocyst. Quddus [22] reported that EBN reduces the level of ROS in the Cd-treated group. This will explain the quality enhancement of the blastocysts in either of the treatment groups, Cd 5 mg/kg BW + EBN 120 mg/kg BW or EBN 120 mg/kg BW alone. Coupled with the lowest pregnancy success rates noted in the recipient group, Cd 5 mg/kg BW + EBN120 mg/kg BW, this can be mainly related to the ability of the Cd to accumulate in blastocysts, resulting in degeneration and decompaction post-formation. It has been pointed out that this process involves apoptosis and/or breakdown in cell adhesion by Thompson [34]. According to Albishtue [17], deposition of Cd in the blastocyst may decrease embryo attachment capability to the endometrium. These results are consistent with our observation, as there was a low percentage of pregnancy in the T4 group treated with Cd 5 mg/kg BW + EBN 120 mg/kg BW and no pregnancy in the groups T1, T5, and T6. These findings confirm that EBN can prevent the adverse effect of Cd on pregnancy.

## Conclusion

EBN at 120 mg/kg bw led to higher blastocyst production and better quality compared to Cd-exposed groups. The highest pregnancy rates in recipient groups correlated with the highest blastocyst scores from donors. EBN at 120 mg/kg demonstrated significant protection against Cd toxicity and its effect on pregnancy rates, embryo production, quality, and pre- and post-ET, surpassing the effects of both 90 mg/kg EBN and EDTA.

## List of abbreviations

BW, Body weight; Cd, Cadmium; EBN, Edible bird’s nest; ET, Embryo transfers.
